# Molecular characterization of a novel mosaic *tet*(S/M) gene encoding tetracycline resistance in foodborne strains of *Streptococcus bovis*

**DOI:** 10.1099/mic.0.058206-0

**Published:** 2012-09

**Authors:** Simona Barile, Chiara Devirgiliis, Giuditta Perozzi

**Affiliations:** INRAN – National Research Institute on Food & Nutrition, Via Ardeatina 546, 00178 Roma, Italy

## Abstract

The presence of antibiotic-resistance (AR) genes in foodborne bacteria of enteric origin represents a relevant threat to human health in the case of opportunistic pathogens, which can reach the human gut through the food chain. *Streptococcus bovis* is a human opportunistic pathogen often associated with infections in immune-compromised or cancer patients, and it can also be detected in the environment, including fermented foods. We have focused on the molecular characterization of a tetracycline (Tet)-resistance gene present in 39 foodborne isolates of *S. bovis* phenotypically resistant to this drug. The gene was identified as a novel *tet*(S/M) fusion, encoding a mosaic protein composed of the N-terminal 33 amino acids of Tet(S), in-frame with the Tet(M) coding sequence. Heterologous expression of the mosaic gene was found to confer Tet resistance upon *Escherichia coli* recipients. Moreover, the *tet*(S/M) gene was found to be transcriptionally inducible by Tet under the endogenous *tet*(S) promoter in both *S. bovis* and *E. coli.* Nucleotide sequencing of the surrounding genomic region of 16.2 kb revealed large blocks of homology with the genomes of *Streptococcus infantarius* and *Lactococcus lactis.* A subregion of about 4 kb containing mosaic *tet*(S/M) was flanked by two copies of the IS*1216* mobile element. PCR amplification with primers directed outwards from the *tet*(S/M) gene identified the presence of a 4.3 kb circular form corresponding to the intervening chromosomal region between the two IS*1216* elements, but lacking a replication origin. The circular element shared extensive overall homology with a region of the multidrug-resistance plasmid pK214 from *Lc. lactis*, containing *tet*(S), as well as the IS*1216* transposase-containing element and intervening non-coding sequences. Linear reconstruction of the insertion events likely to have occurred within this genomic region, inferred from sequence homology, provides further evidence of the chromosomal rearrangements that drive genomic evolution in complex bacterial communities such as the gut and food microbiota.

## Introduction

*Streptococcus bovis* is an indigenous resident of the gastrointestinal (GI) tract in humans and animals. It is classified as a member of the group D streptococci, which includes the highly related species *Streptococcus equinus*, *Streptococcus caprinus*, *Streptococcus gallolyticus*, *Streptococcus galactolyticus*, *Streptococcus infantarius*, *Streptococcus macedonius* and *Streptococcus waius* (Herrera *et al.*, 2009). Several *Streptococcus* species represent serious invasive pathogens, often associated with wound infections, sepsis, abscesses and dental caries in immunocompromised or cancer patients (Moet *et al.*, 2007; Fernández-Ruiz *et al.*, 2010; Al-Jashamy *et al.*, 2010). The association of *S. bovis* with endocarditis (Gupta *et al.*, 2010), colon cancer (Boleij *et al.*, 2009) and colon adenoma (Kahveci *et al.*, 2010) has also recently been reported. This species is therefore considered a potential pathogen.

The widespread use of antibiotics has applied strong selective pressure in the environment, favouring the survival and spread of antibiotic-resistant (AR) bacterial species. Such environmental selection is reflected in the increasing presence of AR commensal bacteria in the gut microbiota of livestock, which in turn leads to an increased frequency of AR species within the microbiota of fermented foods of animal origin, such as dairy and meat products. AR commensal bacteria do not by themselves represent a threat to human health, but their presence in the gut microbiota of humans and animals is increasingly viewed as a reservoir with the potential of being transmitted to pathogens through genomic exchange (Ammor *et al.*, 2007). This is especially relevant for opportunistic pathogens, which are also capable of acquiring virulence genes and are reported to be important agents of AR nosocomial infection. The key role played by mobile elements in AR gene transfer was further emphasized by the publication of the first sequenced genome of a vancomycin-resistant clinical isolate of *Enterococcus faecalis*, revealing the presence of up to 38 insertion sequences (ISs) (Paulsen *et al.*, 2003). Transposon-mediated inter-species transfer of AR genes has now been further characterized as one of the main mechanisms contributing to AR spread in bacteria (Wozniak & Waldor, 2010).

In the present work we report the molecular characterization of a novel functional mosaic tetracycline (Tet)-resistance gene that was identified in foodborne isolates of *S. bovis*. We also studied the genomic context surrounding the mosaic gene, whose sequence shares long stretches of homology with the genomes of other streptococcal species.

## Methods

### 

#### Bacterial strains and growth conditions.

*S. bovis* strains were cultured in de Man, Rogosa and Sharpe (MRS) medium. Growth conditions and antibiotic concentrations were as previously described (Devirgiliis *et al.*, 2010).

#### Cloning procedures.

A 2603 bp DNA fragment containing full-length mosaic *tet*(S/M) and the 5′ and 3′ untranslated regions was amplified using the TetS/M-F-*Sal*I and TetS/M-R-*Pst*I primers (Table 1). The purified PCR product was digested with *Sal*I and *Pst*I and cloned into pBluescript KS+ (Stratagene), linearized with the same enzymes. The resulting construct was used to transform electro-competent *Escherichia coli* DH5α.

**Table 1.** Primers used in PCR amplifications Nucleotides corresponding to restriction site sequences are in italic type. Primers marked with ‘×’ in the Walking primer column were also used for gene walking.

Primer	Sequence (5′–3′)	Target region	Annealing temperature (°C)	Walking primer	Source or reference
TetS/M-F-*Sal*I	gggccc*GTCGAC*TAGCCATTCTGAAGAGTTATCTTC	−379ATG *tet*(S)	64		This work
TetS/M-R-*Pst*I	gggccc*CTGCAG*CGAATTATCGGCTCTGCGTCTTTGC	Orf6 stop codon	64		This work
STRINF2-REV	GGCATTCTCCAATATGTGGAGC	STRINF_00828	62	×	This work
IS1068-FW	CGTCGTCCTTCAAAACAACAAGTGG	llkf_p0022	62	×	This work
KUP-FW	CGTTTAATAAGGCCACGAGTGCAGG	llkf_p0020	62	×	This work
TetS-1-FW	GGCTATCAGTGTGTAGTAGTATACAGC	*tet*(S) promoter	65		This work
TetS-2-REV	GCTGTATACTACACACTGATAGCCACAGATGC	*tet*(S) promoter	68	×	This work
Rev-Ext	GCTTTCCTCTTGTTCGAGTTCCAATGC	*tet*(M)	68	×	Devirgiliis *et al.* (2009)
For-Ext	CATTCACATCGAAGTGCCGCCAAATCC	*tet*(M)	68		Devirgiliis *et al.* (2009)
Tet-int-FW	CGGATAGATAAAGTACGATA	*tet*(M)	54	×	Devirgiliis *et al.* (2009)
Orf6-FW	GTGGATATTGTGTCCTGTATGTGG	Orf6	58	×	This work
STRINF1-REV	GGAGTTTTGCTTCTCATACTTGGGG	STRINF_00831	62	×	This work

#### DNA extraction and molecular analysis.

Genomic DNA was extracted using the MagPrep Bacterial Genomic DNA kit (Merck), according to manufacturer’s instructions. PCR amplifications were performed as previously described (Devirgiliis *et al.*, 2008), with the primers listed in Table 1 (Primm, Italy). PCR products were purified with the NucleoSpin Extract II purification kit (Macherey-Nagel), and sequenced by the M-Medical sequencing service (Italy). Southern hybridizations were carried out using standard protocols, with probes labelled with digoxigenin-11-dUTP (Roche Diagnostics). Restriction endonucleases were purchased from Promega.

#### RT-PCR.

Bacterial RNA was isolated using TRIzol reagent (Invitrogen) according to the manufacturer’s instructions and subsequently treated with DNase I (RNase-free). One microgram of total RNA was reverse-transcribed with M-MLV reverse transcriptase (Invitrogen) in a final volume of 20 µl, using random hexamers as primers. Two microlitres of the reaction mix was then used as template in PCRs with primers amplifying either the 16S rDNA (control) or the *tet*(S/M) mosaic gene (primers TetS-1-FW and Rev-Ext; Table 1).

#### Gene walking.

The two-step gene walking method consisted of a walking-PCR (step 1) followed by direct sequencing of the PCR product (step 2), as described in (Pilhofer *et al.*, 2007). Walking-PCRs were performed in a final volume of 50 µl containing 0.5 µM of the specific primer (Table 1), 2.5 U AccuTaq (Sigma), 1× AccuTaq buffer, 2.5 mM of each dNTP and 50 ng template DNA. The cycling program was the same as that reported in (Pilhofer *et al.*, 2007), except for the annealing temperature of specific primers (see Table 1) and the AccuTaq extension temperature (68 °C). Using this method, overlapping DNA fragments ranging from 3 to 4 kb were obtained with walking primers directed outwards, upstream of *tet*(S/M) (Rev-Ext, TetS-2-REV, KUP-FW, IS1068-FW, STRINF2-REV in Table 1). The sequence of the region downstream of *tet*(S/M) was obtained following three rounds of walking with primers Tet-int-FW, Orf6-FW and STRINF1-REV (Table 1). Walking-PCR products were purified using a NucleoSpin Extract II kit according to the manufacturer’s instructions and were fully sequenced. Computer-assisted joining of the sequenced fragments to obtain the complete sequence was performed using the web resource CLC Sequence Viewer 6.0.2 (www.clcbio.com).

## Results

### Identification of a novel *tet*(S/M) mosaic gene

**Fig. 1.**
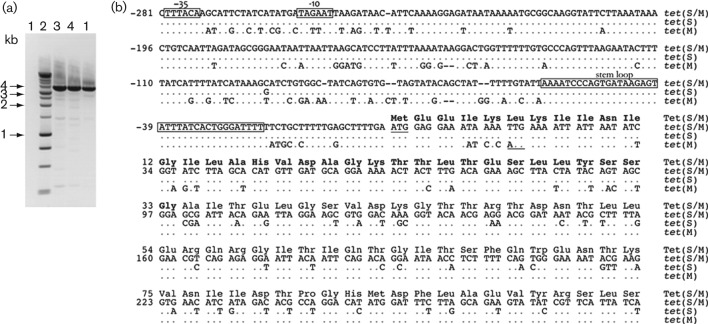
Amplification and sequence analysis of the mosaic *tet*(S/M) gene. (a) PCR amplification with primers directed outwards from *tet*(S/M). Lanes: 1, no DNA control; 2, size marker (1 kb ladder); 3–5, template DNA from *S. bovis* independent isolates (1315, 1357 and 1400; Devirgiliis *et al.*, 2010). (b) Sequence alignment of mosaic *tet*(S/M) with *tet*(S) (accession no. X92946) and *tet*(M) (U09422), including the portion of coding sequence where fusion occurred between the two *tet* genes and the untranslated regulatory region upstream of the *tet*(S) ATG. Transcriptional and translational regulatory sequences (annotated in X92946) are boxed. Mosaic *tet*(S/M) and *tet*(M) ATG start codons are underlined. Amino acid residues contributed by Tet(S) in the mosaic protein are shown in bold type.

**Fig. 2.**
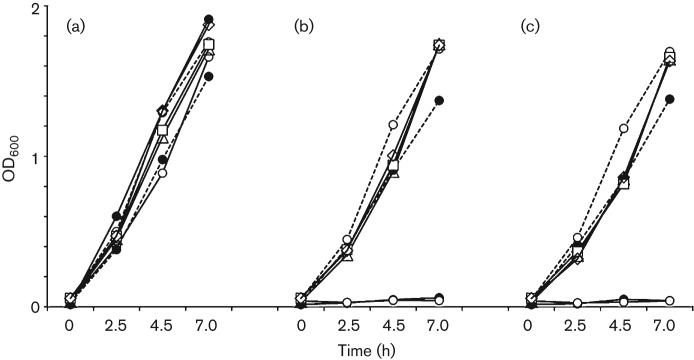
Heterologous expression of Tet(S/M) in *E. coli* confers Tet resistance. Growth curves of three independent recombinant colonies of *E. coli* strain DH5α expressing *S. bovis tet*(S/M) in the absence (a) or presence of Tet at 6 mg l^−1^ (b) and 12 mg l^−1^ (c). The three transformants are labelled with □, ▵ and ◊. Negative controls: *E. coli* strain DH5α (•), strain DH5α with empty vector (○); positive controls: *E.* *coli* strain XL1-Blue with empty vector (○, dashed line) or with *S. bovis tet*(S/M) (•, dashed line).

The Tet-resistance determinant in phenotypically resistant foodborne strains of *S. bovis* isolated in our laboratory was previously reported as *tet*(M) on the basis of PCR amplification with *tet*(M)-specific primers (Devirgiliis *et al.*, 2010). To further characterize the resistance gene, three representative isolates belonging to different repetitive extragenic palindromic (rep) groups were analysed at the molecular level. Since *tet*(M) is most commonly found within mobile elements in Gram-positive bacteria (Flórez *et al.*, 2008), the PCR strategy chosen used primers directed outwards from the *tet*(M) gene (Table 1, For-Ext and Rev-Ext). These primers could yield an amplicon only in the case of a circular template. This approach yielded a 3.7 kb amplicon (Fig. 1a), which was sequenced fully, revealing a novel Tet-resistance gene represented by a *tet*(S)*–tet*(M) fusion. The DNA sequence of the relevant portion of the mosaic *tet* gene is shown in Fig. 1(b) with the corresponding primary sequence of the protein, consisting of the amino-terminal 33 amino acids of Tet(S), fused to Tet(M) residues 28–639. To ensure that the mosaic gene was indeed responsible for conferring phenotypic resistance to Tet upon the *S. bovis* isolates, we first tested for the possible presence of other Tet-resistance determinants by PCR and/or by Southern blotting. With these approaches, we could exclude the presence of the most commonly occurring *tet* resistance genes in Gram-positive bacteria: *tet*(O), *tet*(S), *tet*(L), *tet*(K) and *tet*(W) (data not shown). We then tested the ability of the mosaic *tet*(S/M) gene to confer Tet resistance by heterologous expression in *E. coli.* A 2.6 kb fragment containing the entire *tet*(S/M) ORF, with long stretches of 5′ and 3′ untranslated sequences, was cloned into the plasmid vector pBluescript KS+, and the resulting construct was transformed into the Tet-sensitive *E. coli* DH5α strain. Three independent recombinant colonies were tested for their ability to grow in the presence of Tet in liquid culture, using the MIC for *E. coli* cells in Tet as a reference concentration (5 mg l^−1^). As shown in Fig. 2, *E. coli* transformants expressing *S. bovis tet*(S/M) were able to survive at the maximum Tet concentration tested (12 mg l^−1^), with growth curves displaying the same slope as the Tet-resistant *E. coli* strain XL1Blue, which harbours the Tn*10* transposon and is therefore intrinsically resistant to Tet.

### Genomic localization of *tet*(S/M)

**Fig. 3.**
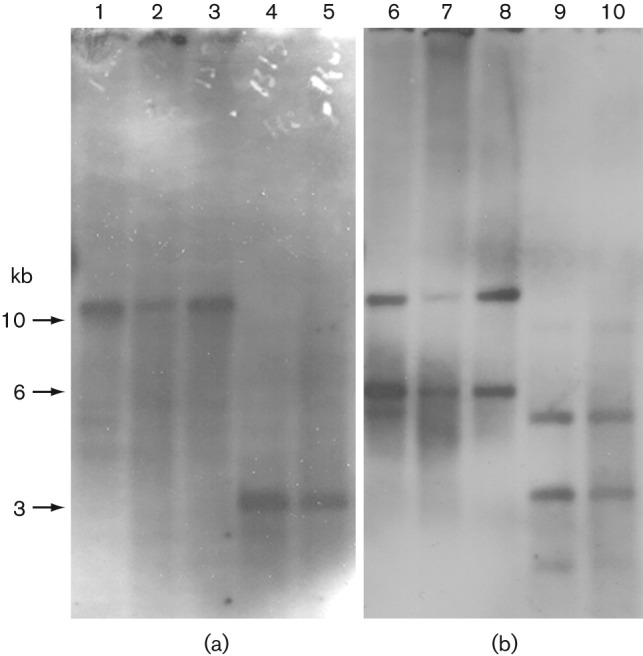
Genomic localization of the *tet*(S/M) gene. Southern blot analysis of *Eco*RI- and *Hin*dIII-digested genomic DNA from independent isolates of *S. bovis* probed with *tet*(M) (a) and *tnpA* (b) gene fragments. Lanes 1–3 and 6–8, *Eco*RI-digested DNA from isolates 1315, 1357 and 1400; lanes 4–5 and 9–10, *Hin*dIII-digested DNA from isolates 1315 and 1357.

To begin analysing the genomic context of the newly identified resistance gene, we extracted genomic DNA from the three representative *S. bovis* strains, and subjected it to Southern blot analysis. Results in Fig. 3 show that *tet*(S/M) is present in single copy within a genomic *Eco*RI fragment larger than 10 kb and a *Hin*dIII fragment of 3 kb, specifically hybridizing with the *tet*(M) probe (Fig. 3a). A *tnpA* probe was used as an internal control, because sequencing of the 3.7 kb amplicon had shown the presence of an IS belonging to the IS*1216* family (Mahillon & Chandler, 1998). This probe hybridized with the same genomic *Eco*RI and *Hin*dIII fragments recognized by the *tet*(M) probe, but also with other fragments, indicating the presence of additional ISs in the genome of *S. bovis* (Fig. 3b).

**Fig. 4.**
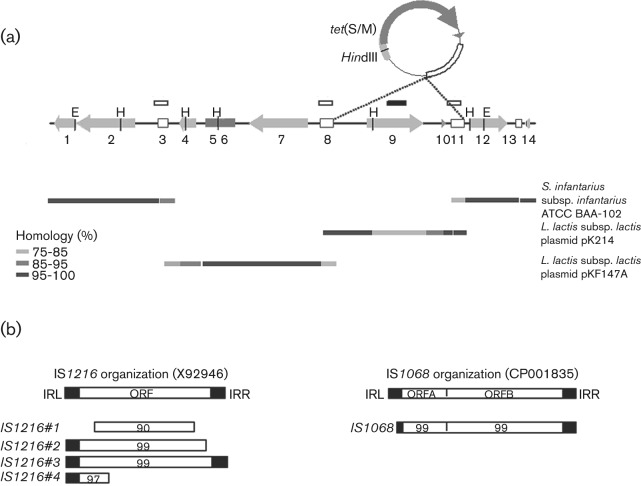
Genomic organization of the *S. bovis* 16 224 bp region flanking the mosaic *tet*(S/M) gene. (a) Schematic representation of the entire sequenced region. The ORFs are represented by arrows oriented in the direction of transcription. Open boxes represent IS*1216*-like transposases, while the dark-grey box corresponds to the IS*1068*-like transposase. *Hin*dIII sites (H) and *Eco*RI sites (E) giving rise to the fragments detected by Southern hybridization are marked. Sequences hybridizing with the *tet*(M) and *tnpA* probes are indicated above the genomic structure by closed and open boxes, respectively. Correspondence between the integrated *tet*(S/M)-containing subregion and the PCR-amplified circular form is indicated by dotted lines. Bars of different grey tones below the linear reconstruction of the entire region indicate homologies with the genomes of sequenced bacterial strains. Gaps between bars represent non-contiguous tracts of homology. (b) Schematic comparison between the IS*1216*-like and IS*1068*-like ISs identified in *S. bovis* and their corresponding reference sequences (accession nos X92946 and CP001835, respectively). Black rectangles represent left (IRL) and right (IRR) inverted repeat sequences; white rectangles indicate transposase ORFs. Numbers within rectangles indicate the percentage nucleotide sequence homology.

**Fig. 5.**
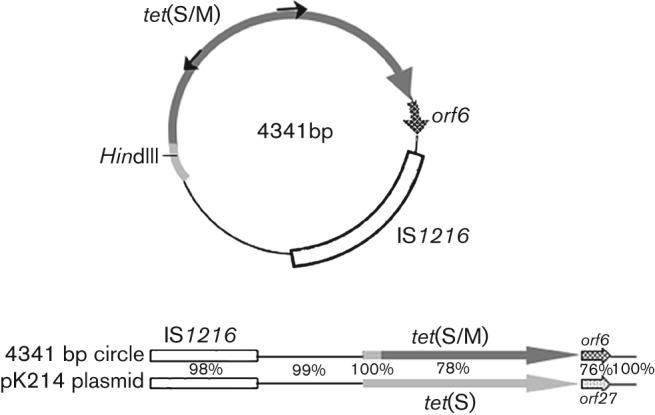
Molecular structure of the 4341 bp circular form containing mosaic *tet*(S/M). Light grey identifies *tet*(S) ORF sequences in the mosaic gene, dark grey highlights *tet*(M) sequences. Sequence comparison between the *tet*(S/M)-containing circle and nucleotides 25513–29826 of *Lc. lactis* plasmid pK214 (accession no. X92946) is shown below the circle. Percentage homology is shown for each coding and non-coding subregion.

Since the *S. bovis* genome has not been fully sequenced, we sought to extend sequence information in the chromosomal region containing *tet*(S/M) using the two-step gene-walking method of Pilhofer *et al*. (2007) (see Methods). Oligonucleotide primers corresponding to both termini of *tet*(S/M) were first synthesized, and then used in PCRs to extend into the surrounding sequence in both directions. As more DNA sequence was determined, the same steps were employed to ‘walk’ further into the genome on both sides. The resulting sequence of a chromosomal region of 16 244 nt is schematically represented in Fig. 4(a). Major observations are as follows: a chromosomal subregion containing the *tet*(S/M) gene is flanked by IS*1216* sites, suggesting that excision events could lead to a 4.3 kb circular form. The results of our previous Southern hybridizations were confirmed at the sequence level, as the position of *Hin*dIII restriction sites identified the following digestion products: (1) the 3 kb *Hin*dIII fragment detected by both *tet*(S/M) and *tnpA* probes (Fig. 3, lanes 4–5, 9–10); and (2) the 5 kb fragment detected by the *tnpA* probe alone (Fig. 3b, lanes 9–10). The remaining sequence showed the presence of three additional ISs, two of them sharing homology with IS*6* family members (IS*1216*), and one with IS*3* family members (IS*1068*) (Mahillon & Chandler, 1998) (Fig. 4b). All IS transposase-encoding ORFs appeared to be truncated by premature stop codons, with the exception of ORF B within IS*1068* (Fig. 4b). Overall, blast searches for sequence similarity showed that this region contains stretches of homology to the pkF147A plasmid of *Lactococcus lactis* subsp. *lactis*, strain KF147 (GenBank accession no. CP001835), and with a chromosomal region of *S. infantarius* subsp. *infantarius*, strain ATCC BAA-102 (ABJK02000017) (Schlegel *et al.*, 2000) (Fig. 4a, Table 2). Noteworthily, the hybrid nature of this region was further suggested by the presence, immediately downstream of the *tet*(S/M) gene, of an ORF deriving from the broad-host-range conjugative transposon Tn*916* (*orf6*, sharing 87 % homology with *orf27* of *Lc. lactis* pK214) (Fig. 5).

**Table 2.** List of known *S. infantarius* and *Lc. lactis* genes homologous to the ORFs detected in the sequenced region of the *S. bovis* genome depicted in Fig. 4

ORF no.	Gene name or locus tag	Protein	Putative function	DNA homology (%)	Amino acid identity (%)	Accession no.	Reference
1	STRINF_00827	Hypothetical protein	ATPase component (ABC-type uncharacterized transport system)	99	99	ABJK02000017	
2	STRINF_00828	Hypothetical protein	Permease component (ABC-type uncharacterized transport system)	99	99	ABJK02000017	
3	*tnpA*	IS*1216* transposase A portion (77–152 aa)	Transposase	90	96	X92946	Perreten *et al.* (1997)
4	LLKF_p0016	Resolvase	Resolvase	92	97	CP001835	Siezen *et al.* (2010)
5	LLKF_p0022	IS*1068* transposase (C terminus)	Transposase	99	98	CP001835	Siezen *et al.* (2010)
6	LLKF_p0021	IS*1068* transposase (N terminus)	Transposase	96	88	CP001835	Siezen *et al.* (2010)
7	LLKF_p0020	Potassium transport system protein kup 1	Potassium transporter	99	99	CP001835	Siezen *et al.* (2010)
8	*tnpA*	Truncated IS*1216* transposase A (1–105 aa)	Transposase	99	100	X92946	Perreten *et al.* (1997)
9	*tet* (S/M)	Tet(S/M)	Tet resistance	79	77	X92946	Perreten *et al.* (1997)
10	*orf6*	Hypothetical protein	Unknown function	87	81	X92946	Perreten *et al.* (1997)
11	*tnpA*	Truncated IS*1216* transposase A (1–105 aa)	Transposase	99	100	X92946	Perreten *et al.* (1997)
12	STRINF_00831	Hypothetical protein	Catalytic activity (polysaccharide deacetylase domain)	99	98	ABJK02000017	
13	*tnpA*	N-terminal fragment IS*1216* transposase A (1–34 aa)	Transposase	94	88	X92946	Perreten *et al.* (1997)
14	STRINF_00836	Hypothetical protein	Regulator of disulfide bond formation	99	99	ABJK02000017	

### Tet induction of the mosaic *tet*(S/M) gene

**Fig. 6.**
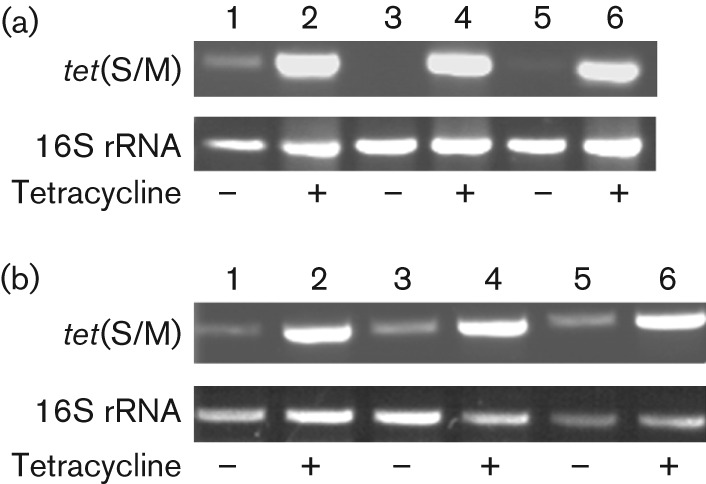
Tet-dependent induction of *tet*(S/M) expression. RT-PCR analysis of mosaic *tet*(S/M) gene expression in: (a) three independent *S. bovis* isolates grown in the presence (lanes 2, 4 and 6) or absence (lanes 1, 3 and 5) of Tet (8 mg l^−1^); (b) three independent *E. coli* DH5α transformants expressing *S. bovis* Tet(S/M), grown in the presence (lanes 2, 4 and 6) or absence (lanes 1, 3 and 5) of Tet (12 mg l^−1^). Reverse-transcribed 16S rRNA was used as an internal control.

Tet is well known to induce transcription of the corresponding resistance genes in Gram-negative bacteria (Berens & Hillen, 2003). Tet-dependent regulation of *tet* gene expression in Gram-positive organisms has been shown to be associated with increased transposition frequency of Tn*916* in *Ent. faecalis* and *Bacillus subtilis*, by a mechanism involving transcriptional attenuation (Su *et al.*, 1992; Celli & Trieu-Cuot, 1998; Roberts & Mullany, 2009). We have also reported transcriptional induction of *tet*(M) expression in foodborne strains of *Lactobacillus paracasei* (Comunian *et al.*, 2010), and we therefore sought to analyse expression of the *tet*(S/M) mosaic gene in *S. bovis* strains, where transcription is driven by the *tet*(S) promoter. To this aim, total RNA was extracted from the Tet-resistant *S. bovis* strains, grown in the presence or absence of the antibiotic, and subjected to RT-PCR with specific primers mapping on the *tet*(S) and *tet*(M) sequences (TetS-1-FW and Rev-Ext in Table 1). As shown in Fig. 6(a), *tet*(S/M) expression was very low in the absence of Tet in all three strains examined, while it was strongly induced following antibiotic addition to the growth medium at a final concentration of 8 mg l^−1^. Previously described *E. coli* transformants expressing an *S. bovis tet*(S/M) construct (Fig. 2) were also tested for induction by growth in the presence or absence of 12 mg Tet l^−1^. The results of RT-PCR assays in Fig. 3(b) show that Tet-dependent induction of the mosaic gene, driven by the *S. bovis tet*(S) promoter, could also be retained in the *E. coli* heterologous expression system, suggesting that proteins involved in Tet-dependent regulation of gene expression are well conserved in Gram-negative and Gram-positive bacteria.

### Identification of an intermediate circular form

As described above, amplification of the region containing mosaic *tet*(S/M) was achieved using diverging primers on the *tet*(M) sequence that could yield an amplicon only in the presence of a circular template. Furthermore, sequence analysis of the genomic context of the newly identified resistance gene revealed that it localizes within a 4.3 kb chromosomal subregion delimited by ISs, suggestive of potential excision and circularization events. However, no sequence resembling that of a replication origin was identified within the entire 4.3 kb region, suggesting that an excised circle containing *tet*(S/M) would be unable to sustain autonomous replication. The results of Southern blot hybridization in Fig. 3 seem to confirm the absence of detectable levels of the circular form, as the 4.3 kb *Hin*dIII band predicted from the DNA sequence as a linearization product of the circular form could not be detected with either *tet*(M) or *tnpA* probes. However, since we were indeed able to amplify a *tet*(S/M)-containing circle by PCR (Fig. 1a), this result suggests the low abundance or transient nature of the circle. blast sequence similarity searches with the genomic region containing the mosaic *tet*(S/M) gene revealed homology with a region of *Lc. lactis* plasmid pK214 (GenBank accession no. X92946, nucleotides 25513–29826), carrying the Tet-resistance gene *tet*(S) and the IS*1216* mobile element (Perreten *et al.*, 1997) (Fig. 5). However, the DNA sequence of the transposase-encoding *tnpA* gene within the IS*1216* element of *S. bovis* contained a nonsense mutation, leading to a translational STOP codon (UGA) at amino acid position 105.

## Discussion

Foodborne bacteria that survive the harsh conditions of the upper GI tract and reach the colon become part of the crowded gut microbiota, thus contributing to its composition, which in turn can affect the health/disease balance of the host (Garrett *et al.*, 2010). Although fermented food products mostly contain probiotic species, they can also contribute a small fraction of bacteria which are not favourable for health, such as opportunistic pathogens capable of acquiring virulence traits in immune-compromised individuals. Among them, enterococci are the most abundant genus found in the human GI tract (Ogier & Serror, 2008), but streptococci are also represented (Herrera *et al.*, 2009). In the latter case, some species have been identified as responsible for common infections (Fernández-Ruiz *et al.*, 2010; Srivastava *et al.*, 2010). Horizontal transfer of AR determinants to pathogens raises growing concern, especially in light of the potential inter-species transferability of AR genes, suggested by their association with mobile elements (Freitas *et al.*, 2011). For this reason it is important not only to identify the occurrence of AR genes in foodborne bacteria but also to characterize their genomic context, with special focus on the regions involved in inter-species and intra-species transfer. Conjugative plasmids containing transposon Tn*1546* carrying the *van*(A) operon seem to have played a relevant role in the recent increase of vancomycin-resistant enterococci (VRE) isolated from poultry and swine sources. Tn*1546* variants are associated with IS*1216* ISs, which are capable of autonomous transposition and greatly contribute to mobilization of flanking genomic sequences (Novais *et al.*, 2008). *Tet*(M) and *tet*(S) have been frequently shown to be in association with the conjugative transposon Tn*916* (Roberts & Mullany, 2009). In the present work, we report the association of a novel mosaic Tet-resistance gene [*tet*(S/M)] with a potentially mobile element closely resembling a region of the *Lc. lactis* plasmid pK214 (Perreten *et al.*, 1997). The gene was identified in a foodborne strain of the opportunistic pathogen *S. bovis* formerly isolated from the fermenting microflora of raw buffalo milk (Devirgiliis *et al.*, 2010). Sequencing of the gene and of the surrounding genomic region revealed its molecular organization as that of a novel mosaic Tet-resistance gene consisting of the *tet*(S) promoter region followed by a small portion of the ORF (encoding 33 N-terminal amino acids) fused in-frame with the *tet*(M) ORF. Notably, the primary sequences of Tet(S) and Tet(M) share an overall degree of homology of 78 %, aside from their difference in length (646 and 639 amino acids, respectively), which is due to the presence of five additional Tet(S)-specific N-terminal amino acids. We demonstrate that the mosaic *tet*(S/M) gene is functional, as it was able to confer Tet resistance by heterologous expression in *E. coli* under the control of the cloned *tet*(S) promoter. Tet-dependent transcriptional induction of the mosaic gene, which we demonstrated in *S. bovis*, was retained in the heterologous host. Transcriptional upregulation of *tet* genes was elegantly described in Gram-positive bacteria as an attenuation mechanism dependent on readthrough of small ORFs downstream of the *tet*(M) coding region (Su *et al.*, 1992; Celli & Trieu-Cuot, 1998; Roberts & Mullany, 2009). The sequence of the *S. bovis* mosaic gene does not contain such regulatory ORFs, so it will be extremely interesting to further characterize the mechanism conferring Tet-dependent upregulation upon the *tet*(S) promoter.

The existence of naturally occurring mosaic Tet-resistance genes has previously been reported in other genera (Stanton *et al.*, 2005; Patterson *et al.*, 2007; van Hoek *et al.*, 2008), but to the best of our knowledge this is the first example of a chimera involving *tet*(S) and *tet*(M). Southern blot analysis of total DNA extracted from *S. bovis* showed that *tet*(S/M) is present in single copy in the bacterial genome, within a subregion flanked by two IS*1216* elements. This region can also be amplified by PCR using primers directed outwards from *tet*(S/M), indicating the existence of an extra-chromosomal circular form. Complete sequencing of the PCR amplicon revealed the presence of a single copy of IS*1216*, suggestive of transposase-mediated circularization/excision of the genomic *tet*(S/M)-containing region, occurring at the two flanking IS*1216* sites. However, we could not identify a transfer origin similar to those found in conjugative transposons, and the linearized circle was undetectable in Southern hybridizations. Moreover, *in vitro* conjugal transfer of Tet resistance to an *Ent. faecalis* recipient was unsuccessful (Devirgiliis *et al.*, 2010). All this leads us to speculate that the circular form may represent a transposition intermediate rather than a self-replicating extra-chromosomal element, possibly occurring in only a subset of cells and mobilized only within the same cell. Circular elements often represent transient replication intermediates, typical of transposable elements such as Tn*916* (Clewell *et al.*, 1995). Replication of such elements is promoted by transposases and generates circular molecules, which can either integrate at different chromosomal locations or be horizontally transferred to other cells (Churchward, 2002). The two IS*1216*-like repeats flanking the *S. bovis tet*(S/M) gene might be involved in circularization via homologous recombination. Such non-replicative circular elements harbouring Tet-resistance genes were first described in a *Butyrivibrio fibrisolvens* transconjugant, where two direct repeats flanking *tet*(W) in the donor strain were postulated to give rise to a circular intermediate via homologous recombination (Kazimierczak *et al.*, 2006). IS*1216*-like modules have been reported in association with AR determinants in streptococcal strains. The *tet*(S) gene in Tet-resistant clinical isolates of *Streptococcus dysgalactiae*, as well as the *erm*(T) gene in erythromycin-resistant clinical isolates of *S. gallolyticus*, are both flanked by two copies of IS*1216* in the same orientation (Tsai *et al.*, 2005). In both cases, the structural organization reported for the sequences flanking the AR genes is similar to that of the *tet*(S)-flanking region in pK214.

Sequencing of a longer, 16 kb genomic region surrounding the mosaic AR gene shows the presence of several ISs, suggestive of a mobilizable region. Although most of the ORFs encoding transposases appear to be truncated, we cannot exclude that some of them might be functional, as evidenced by the presence of the amplifiable *tet*(S/M)-containing circular form. Notably, it has been reported that C-terminal-truncated transposases play a role in the reduction of transposition activity (Gueguen *et al.*, 2006). Transposase inactivation appears to be a common feature of IS elements, and is recognized as a regulatory mechanism (Mahillon & Chandler, 1998).

The *S. bovis* genome has not yet been fully sequenced, and very little information is available in databases. The closest species whose entire genome sequence is available is *S. infantarius* (accession no. ABJK02000000). The taxonomic status of *S*. *bovis* strains has been evolving in the past few decades and has progressively changed in parallel with the characterization of new species originally described as *S*. *bovis* (Facklam, 2002). Our Tet-resistant foodborne isolates belong to the *S. bovis–equinus* complex, which includes different species and subspecies isolated from infected humans or animals (Farrow & Collins, 1984; Schlegel *et al.*, 2003). The *S. bovis–equinus* complex is divided into four DNA clusters, one of which consists of *S. infantarius* subsp. *coli* and subsp. *infantarius*. The latter subspecies has been isolated from foodstuffs and from infected humans, where it was found to be associated with systemic disease in infants (Bouvet *et al.*, 1997; Schlegel *et al.*, 2000, 2004). Due to their association with several human and animal diseases, as well as their occurrence in fermented foods, we believe that it is extremely important to determine the genomic sequences of the most widespread species belonging to the *S. bovis–equinus* complex, which will serve as a basis to identify the genetic determinants in infectious strains as well as their potential association with AR determinants.

We take these results as strongly indicative of extensive genomic exchanges, possibly through conjugative events, between species that are natural components of the human microbiota, and which should be more thoroughly investigated in light of their potential role as disease-causing agents.
